# Supplementary material for: Impact of Preharvest and Postharvest on Color Changes during Convective Drying of Mangoes
Source: Foods. 2021 Feb 25;10(3):490. doi: 10.3390/foods10030490 (PMC7996146; doi:10.3390/foods10030490)
Supplement: Supplementary file 1 [file foods-10-00490-s001.zip › foods-1099986-supplementary.pdf]

foods

## Supplementary Material

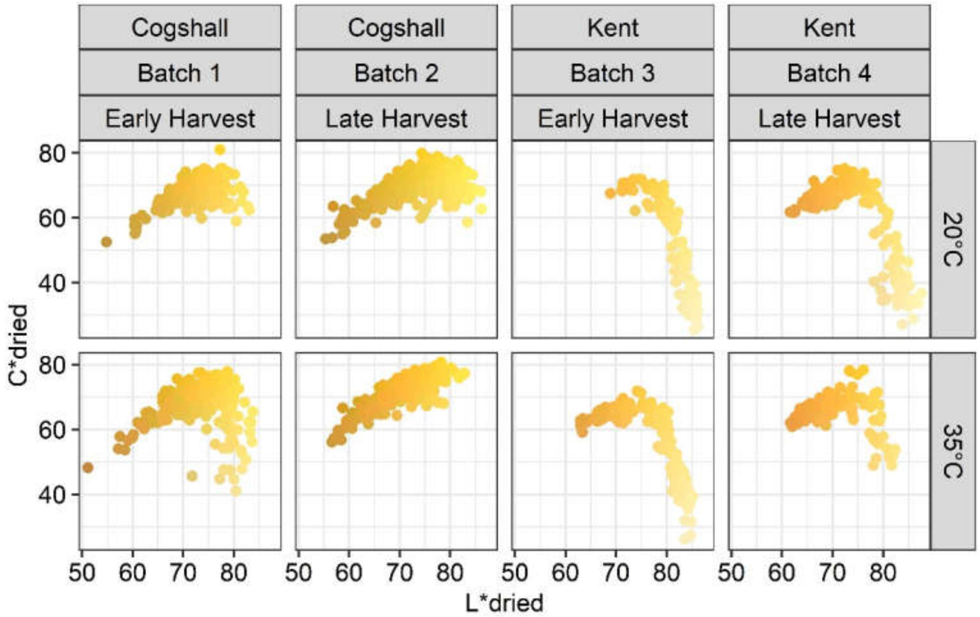


**Figure S1.** Relationships between C* and L* from color measurements on dried mangoes according Table 10. H* and C* coordinates into RGB.
